# A Testator's Intention

**Published:** 1915-06-05

**Authors:** 


### A TESTATOR'S INTENTION.

One would think that, of all causes, a subject like anti-vivisection would gain by unity and concentration. A recent case regarding a legacy bequeathed for the cause discloses the number and diversity of agencies which are furthering it. A Scottish lady directed her trustees to "hold a sum of £1,800 in trust to be devoted by them specially towards the total and absolute prohibition of vivisection." The trustees, owing to differences among themselves on the subject of vivisection, were unable to arrange for the expenditure of the money at their own hand; they applied to the Court to settle a scheme for the disposal of the money, and thereupon four claimants to the fund appeared: (1) the British Union for the Abolition of Vivisection, (2) the Scottish Society for the Total Suppression of Vivisection, (3) the National Anti-Vivisection Society, and (4) the Scottish Society for the Prevention of Vivisection. We need not here go into the arguments by which each of these societies maintained that they should receive the legacy, but simply note the result. The Court proceeded on the intention of the testator so far as they could gather it from her actions when in life, and selected the Scottish Society for the Total Suppression of Vivisection as the legatee intended, on the grounds that the deceased had actively supported it, that it is a Scottish society, and that it advocates prohibition absolutely, and not restrictive measures as well. The following passage from the opinion of one of the judges shows how they inferred the testator's mind on the last point: "The National Anti-Vivisection Society also claims the testatrix as a member on account of her connection with the 'Victoria Street Society for the Protection of Animals from Vivisection,' founded in 1785. About 1897 the Victoria Street Society was reconstituted as the National Anti-Vivisection Society, but a number of the supporters of the Victoria Street Society, including the founder, Miss Frances Power Cobbe, refused to support the reconstituted society, and founded the British Union for the Abolition of Vivisection, on the ground that the efforts of the reconstituted society were not to be confined, as the efforts of the Victoria Street Society had been, to the total abolition of vivisection, but were to embrace, if not to be mainly directed towards, restrictive measures. There is no satisfactory evidence that the deceased was a member of the reconstituted society, and there is good reason to believe that her sympathies were with those who founded the British Union."

